# A Label-Free Microelectrode Array Based on One-Step Synthesis of Chitosan–Multi-Walled Carbon Nanotube–Thionine for Ultrasensitive Detection of Carcinoembryonic Antigen

**DOI:** 10.3390/nano6070132

**Published:** 2016-07-11

**Authors:** Huiren Xu, Yang Wang, Li Wang, Yilin Song, Jinping Luo, Xinxia Cai

**Affiliations:** 1 State Key Laboratory of Transducer Technology, Institute of Electronics, Chinese Academy of Sciences, Beijing 100190, China; xuhuiren_2012@163.com (H.X.); w_yang89@163.com (Y.W.); wangli2011iecas@163.com (L.W.); ylsong@mail.ie.ac.cn (Y.S.); jpluo@mail.ie.ac.cn (J.L.); 2 University of Chinese Academy of Science, Beijing 100049, China

**Keywords:** multi-walled carbon nanotube, microelectrode array, carcinoembryonic antigen, one-step synthesis, label-free

## Abstract

Carcinoembryonic antigen (CEA) has been an extensively used tumor marker responsible for clinical early diagnosis of cervical carcinomas, and pancreatic, colorectal, gastric and lung cancer. Combined with micro-electro mechanical system (MEMS) technology, it is important to develop a novel immune microelectrode array (MEA) not only for rapid analysis of serum samples, but also for cell detection in vitro and in vivo. In this work, we depict a simple approach to modify chitosan–multi-walled carbon nanotubes–thionine (CS–MWCNTs–THI) hybrid film through one-step electrochemical deposition and the CS-MWCNTs-THI hybrid films are successfully employed to immobilize anti-CEA for fabricating simple, label-free, and highly sensitive electro-chemical immune MEAs. The detection principle of immune MEA was based on the fact that the increasing formation of the antigen-antibody immunocomplex resulted in the decreased response currents and the relationship between the current reductions with the corresponding CEA concentrations was directly proportional. Experimental results indicated that the label-free MEA had good selectivity and the limit of detection for CEA is 0.5 pg/mL signal to noise ratio (SNR) = 3. A linear calibration plot for the detection of CEA was obtained in a wide concentration range from 1 pg/mL to 100 ng/mL (*r* = 0.996). This novel MEA has potential applications for detecting CEA for the research on cancer cells and cancer tissue slices as well as for effective early diagnosis.

## 1. Introduction

Although many efforts have been made to develop managements such as screening, surveillance, prevention and treatment in the past few decades, the distant metastasis and dissemination of cancer remains the major contributor to worldwide mortality [1,2]. It is necessary to develop technology for cancer therapy as well as effective early diagnosis [3,4]. Carcinoembryonic antigen (CEA) is a kind of serum-based tumor marker and is extensively used for the clinical diagnosis of several diseases, such as cervical carcinomas, and pancreatic, colorectal, gastric and lung cancer [5,6]. The sensitive, precise and accurate detection at a low concentration level of CEA in serum samples is very significant in biochemistry research, clinical diagnosis and biomedical exploits [7]. The immunoassay is a common technique for the detection of tumor markers, including electrochemistry-, Raman spectroscopy-, chemiluminescence-, fluorescence-, and surface plasmon resonance-based immunoassay methods [8,9].

Recently, there is a growing interest around the electrochemical immunoassay due to its advantages, including rapid analysis, high sensitivity and a relatively low limit of detection [10,11,12,13]. Generally speaking, an electrochemical biosensor involves a transducer system and bio-sensitive film, which are connected by the chemical interface layer [14]. The sensitivity, specificity, stability and repeatability of the biosensor are mainly based on the performance of the bio-sensitive film. Various novel matrices have been explored and used for fabricating the electrochemical biosensor [15,16]. Among the materials, chitosan (CS) polymers have attracted much interest as suitable matrices due to their abundant amino groups, low toxicity and excellent biocompatibility [17,18]. The film-forming property of CS is used for constructing biosensors and immobilizing bioactive molecules, depending on its solubility and insolubility by adjusting the pH with respect to its pKa [19]. Many redox species have been used to develop electro-active nanocomposites for the application of biosensor fabrication. The typical examples include various redox dyes, such as the Prussian blue (ferric hexacyano ferrate) family, thionine (THI), ferrocene and its derivatives [20,21,22]. There is a growing requirement to synthesize stable electro-active nanocomposites with high sensitivity, good stability and good electrochemical performance through a simple synthetic process [23]. Therefore, the selection of catalytic material is as important as the redox species. Multi-walled carbon nanotubes (MWCNTs) have many advantages as a catalytic material over other materials, including high chemical stability, high tensile strength, being nano-sized, high surface area, excellent stiffness, and high electrical conductivity [24,25]. Additionally, the high effective surface area and reactive functional groups are benefits that allow loading high-density redox species and increasing the electrochemical currents [26,27,28]. Nowadays, microelectrode arrays (MEAs) have become a reliable device for detecting neurotransmitters, genetic information and protein in vivo and in vitro [29,30,31,32,33]. Additionally, the functional characteristics of the MEAs allow real-time detection and stable long-term recordings for cells [34,35].

Herein, we report a novel method for the one step synthesis of CS-MWCNTs-THI hybrid film as the bio-sensitive film to immobilize the CEA antibody for constructing the immune MEAs. The CS-MWCNTs-THI hybrid film was modified onto the microelectrodes by electrochemical deposition. Moreover, the amino groups of CS can offer active sites for the immobilization of anti-CEA. Meanwhile, the synergistic effect of MWCNTs and THI could result in the good electrochemical property of the immune MEAs. The CS-MWCNTs-THI hybrid films are successfully used to fabricate label-free, simple and highly sensitive electro-chemical immune MEAs. The immune MEAs were employed for detecting the concentrations of CEA in human serum samples and exhibited good selectivity.

## 2. Results and Discussion

### 2.1. Principles of the Electrochemical Measurement

**Scheme 1:**
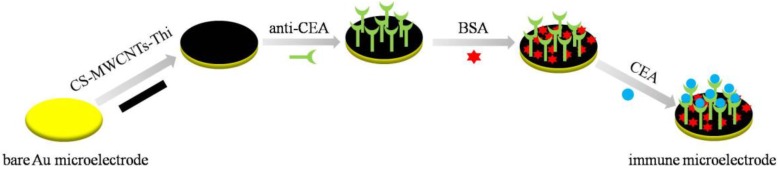
The fabrication procedure of the immune microelectrode array.

The immune MEAs were fabricated using the procedure shown in Scheme 1. The electrochemical performance of CS-MWCNTs-THI hybrid film depended on the synergistic effect between the MWCNTs and THI. The P-type electron clouds of two adjacent atoms overlapping each other formed the π molecular orbital of the THI. The electrocatalytic effect of the MWCNTs was based on its active edge plane with the active functional groups, defect sites and unsatisfied valences. On the basis of the π–π electronic conjugation effect, the overlap of P-type electron clouds reduced the energy barrier for electron transfer between the active sites of the MWCNTs and the THI molecules. The electrocatalytic effect of the MWCNTs and THI increased the current response. Owing to the retardation of the electron transfer process, the formation of the antigen-antibody immunocomplex on the surface of the microelectrodes resulted in the decrease of the current response, and the relationship between the current reductions with the corresponding CEA concentrations was directly proportional. The CEA could be detected quantitatively in serum samples according to this relationship.

### 2.2. Optimization of Microelectrode Preparation

MWCNTs have been widely used as an excellent catalytic material. MWCNTs with favorable conductivity and good biocompatibility are adopted to fabricate electrochemical biosensors for improving electrochemical signals. The lack of MWCNTs resulted in a weak catalytic effect with a low current response of the THI. Excessive MWCNTs were not beneficial for the diffusion of THI and resulted in an increased current baseline. Additionally, size controllability, uniformity and the compactness of the hybrid film were as important as the electrochemical property of the hybrid film. The more negative reaction potential resulted in a high deposition current due to the over-potential accelerating the reaction kinetics of [CS]^+^ deprotonation. Under high deposition current conditions, the hybrid film could cover several microelectrodes with an uncontrolled size. The rapid deposition could increase the roughness of the hybrid film and the uneven surface of the hybrid film resulted in the unstable current response in the cyclic voltammograms (CV) and differential pulse voltammetry (DPV) curves. The excessive MWCNTs could hinder the deposition of CS. It was difficult to form a dense hybrid film with fewer MWCNTs and this kind of hybrid film resulted in the leakage of the THI. The size controllability, uniformity, compactness and electrochemical property should be all considered. The deposition time and reaction potential depended on the composition of the solution and the size of the microelectrode. When the volume ratio of CS and THI was 2:1 and per 300 μL mixed solution contained 2.5 mg MWCNTs, CS could form a uniform and compact matrix to completely cover the single microelectrode. This ratio of THI and MWCNTs also resulted in a stable current signal and good electrochemical response.

### 2.3. Characteristics of the CS-MWCNT-THI Hybrid Film

**Figure 1:**
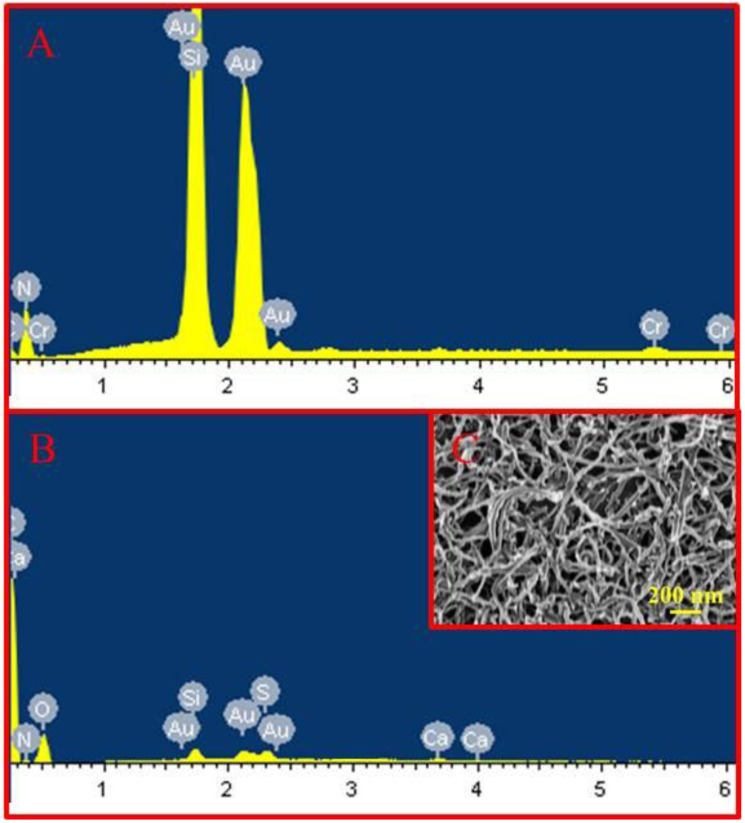
Characteristics of chitosan-multi-walled carbon nanotubes-thionine (CS-MWCNTs-THI) hybrid film. (**A**) Scanning electron microscope (SEM) and energy-dispersive X-ray spectroscopy (EDS) image of bare Au microelectrode; (**B**) SEM-EDS image of CS-MWCNTs-THI hybrid film; (**C**) SEM image of the CS-MWCNTs-THI hybrid film.

Compared with the scanning electron microscope (SEM) energy-dispersive X-ray spectroscopy (EDS) result of the bare Au microelectrode (Figure 1A), the qualitative analysis of SEM-EDS showed the new characteristic peaks of the oxygen element and sulfur element in the CS-MWCNTs-THI hybrid film (Figure 1B). The hydroxyl groups and molecular backbone of CS contributed to the new characteristic peak of the oxygen element and the sulfur characteristic peak derived from the sulfur element of the thionine molecule. These results were proof that CS and THI were embedded into the hybrid film. After modification of the CS-MWCNTs-THI hybrid film, the characteristic peak of the carbon element increased, due to the immobilization of the MWCNTs and CS.

As shown in Figure 1C, the CS-MWCNTs-THI hybrid film displayed s nano-porous structure. This nano-porous structure could increase the effective surface area of the microelectrodes and reduced the impedance, which is beneficial for increasing the current response. In addition, the nano-porous structure promoted the diffusion of electro-active molecules and enhanced the electrochemical diffusion current. The MWCNTs exposed on the surface of the CS-MWCNTs-THI hybrid film were beneficial not only for loading the high-density redox molecules of THI, but also for accelerating the electron transfer of the redox reaction by the electronic conjugation effect, which can enhance electrochemical currents. The nano-porous structure of CS-MWCNTs-THI hybrid film and the synergistic effect of MWCNTs and THI were beneficial for improving the electrochemical current response of the immune MEAs.

### 2.4. Electrochemical Characteristics of the Immune MEAs

**Figure 2:**
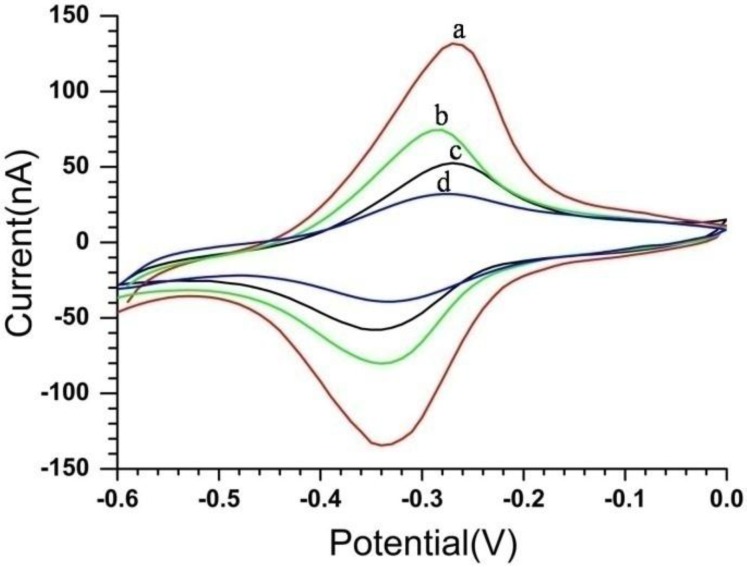
Cyclic voltammograms (CV) of microelectrode modified with CS-MWCNTs-THI/Au (**a**); anti-CEA/CS-MWCNTs-THI/Au (**b**); BSA/CS-MWCNTs-THI/Au (**c**); BSA/anti-CEA/CS-MWCNTs-THI/Au (**d**). Scan rate: 100 mV/s, solution: pH 7.4 phosphate buffer. BSA: bovine serum albumin; CEA: carcinoembryonic antigen.

As illustrated in Figure 2, the modification process of the immune MEAs was monitored by cyclic voltammograms (CVs) in a solution of phosphate-buffered saline (PBS) (pH 7.4). The results of the CVs showed better stability and reproducibility with two pronounced redox peaks. As mentioned before, the immune MEAs first were modified with CS-MWCNTs-THI hybrid film by electrochemical deposition and showed a good electrochemical response with the peak current |*I*_p_| of about 132 nA (as shown in Figure 2, curve a). Consequently, the CS-MWCNTs-THI hybrid film cross-linked anti-CEA by glutaraldehyde (GA), which was employed as the bridging molecule. Owing to the formation of an electron-retarding layer, the loading of anti-CEA resulted in a conspicuous decrease of the peak current and the |*I*_p_| was about 77 nA (Figure 2, curve b). Finally, bovine serum albumin (BSA) solution was used to improve the specificity of immune MEAs by blocking any possible remaining active sites and this BSA layer could avoid the non-specific adsorption. After being blocked with BSA, the current response of immune MEAs further decreased to about 35 nA due to the insulating protein layer of BSA on the microelectrode hindering the electron transfer (Figure 2, curve d). Curve c was the CV of the BSA/CS-MWCNTs-THI/Au microelectrode and the |*I*_p_| was about 52.5 nA. The |*I*_p_| of curve c was lower than the |*I*_p_| of curve b. It was indicated that the BSA could cover the microelectrode more completely than anti-CEA and BSA was used to avoid the contribution of non-specific proteins. Comparing curve c and curve d, the current intensity caused by the anti-CEA in the presence of BSA was about 18.5 nA.

### 2.5. Analytical Performance of the Immune MEAs

**Figure 3:**
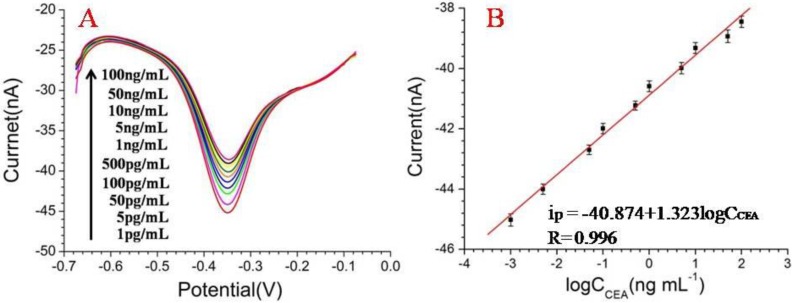
(**A**) Differential pulse voltammetry (DPV) responses of the proposed immune microelectrode arrays (MEAs) after incubation with various concentrations of carcinoembryonic antigen (CEA); (**B**) Calibration curves of the immune MEAs with regard to CEA. Solution: pH 7.4 phosphate buffer.

The electrochemical measurements of DPV were used to investigate the electrochemical response properties corresponding to the adsorption of CEA in the immune MEAs. All the measurements of DPV were carried out in the phosphate buffer (pH 7.4) with the potential range from −0.7 to 0 V. Because the formation of the antibody-antigen immunocomplex on the microelectrodes enhanced steric hindrance and blocked the electron transfer of THI, the peak currents of the DPVs decreased. The concentrations of the corresponding CEA were directly proportional to the current reduction (Figure 3A). Additionally, the calibration plots between the peak current (*I*_p_) and the logarithms of the CEA concentrations are displayed in Figure 3B: *I*_p_ (nA) = −40.874 + 1.323 log *C*_CEA_ (ng/mL) (*r* = 0.996). The results exhibited a good linear detection relationship (LDR) between the peak currents and the logarithms of the CEA concentrations ranged from 1 pg/mL to 100 ng/mL. In addition, the limit of detection (LOD) for CEA by the immune MEAs was 0.5 pg/mL signal to noise ratio (SNR) = 3. The results indicated that the immune MEAs enabled a relatively wide detection range and low LOD for detecting CEA. The relative standard deviation (RSD) of MEAs for the detection of CEA were 11% and 4.9%, respectively, at two concentration levels of 1 ng/mL and 5 ng/mL, which corresponds to the zones of the maximum and minimum errors at the calibration graphs.

### 2.6. Selectivity and Repeatability of the Immune MEA

**Figure 4:**
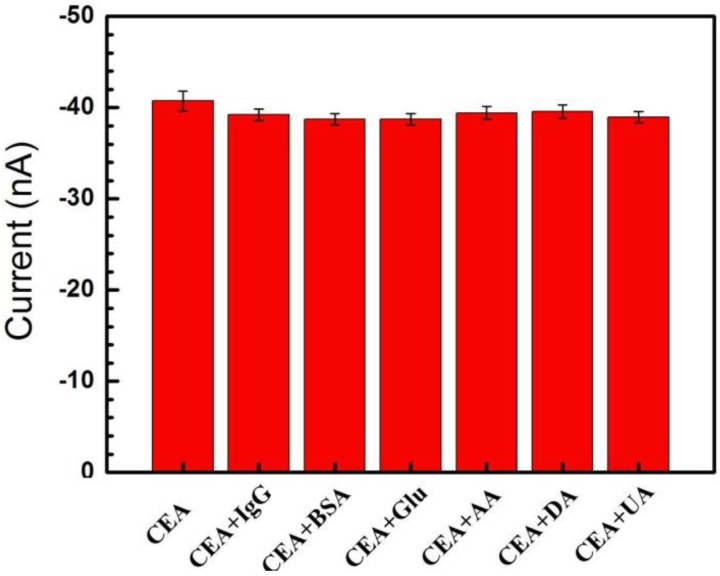
Amperometric response of the immune MEA to 1 ng/mL CEA, 1 ng/mL CEA + 1 ng/mL IgG, 1 ng/mL CEA + 1 ng/mL BSA, 1 ng/mL CEA + 1 ng/mL glucose, 1 ng/mL CEA + 1 ng/mL AA, 1 ng/mL CEA +1 ng/mL DA, 1 ng/mL CEA + 1 ng/mL UA. DA: dopamine; UA: uric acid; AA: ascorbic acid.

In order to evaluate the selectivity of the immune MEAs, some potential interferences were added into 1 ng/mL of CEA, including dopamine (DA), BSA, uric acid (UA), ascorbic acid (AA), glucose (Glu) and human immunoglobulin G (IgG), and the current responses were measured. Compared with the response current of pure CEA (as shown in Figure 4), the variations in the amperometric response caused by the interfering substances were 3.7%, 3.8%, 4.9%, 3.2%, 2.8% and 4.4% (less than 5.0%), respectively, corresponding to the interferences of IgG, BSA, glucose, AA, DA, and UA. The results indicated that the specificity of the proposed immune MEA was acceptably within experimental error and this immune MEA with good selectivity could be employed as an immunosensor system for detecting CEA.

**Figure 5:**
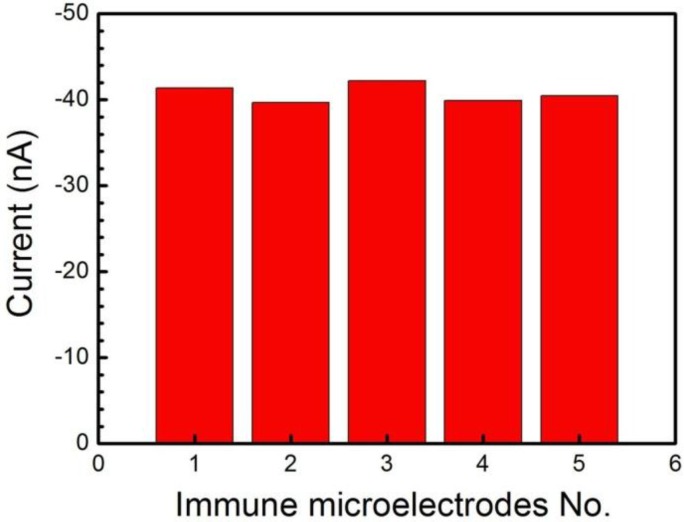
Amperometric responses of five immune microelectrodes on the MEA to 1 ng/mL CEA.

The repeatability of the immune MEAs was also examined by using five equally immune microelectrodes on the MEA for analyzing the same CEA concentration (1 ng/mL) (Figure 5). The five immune microelectrodes, made independently, exhibited similar current responses of 41.38 nA, 39.71 nA, 42.20 nA, 39.88 nA and 40.48 nA, respectively. Therefore, the MEAs exhibited good repeatability with an RSD of 2.6% (less than 5.0%) for the current response of detection for CEA.

### 2.7. Analysis of Clinical Serum Samples

**Figure 6:**
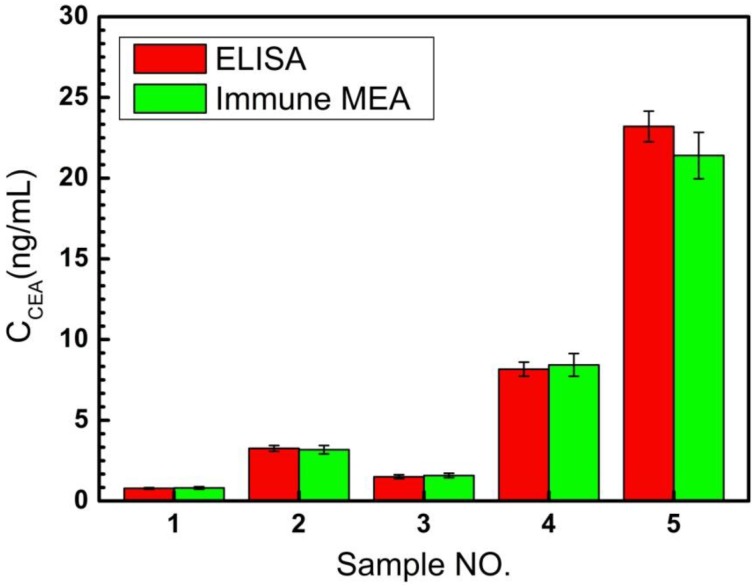
The CEA concentrations of five serum samples detected by enzyme-linked immune sorbent assay (ELISA) and immune MEA.

The recovery tests were performed by adding a standard solution of known concentrations into the diluted serum samples of CEA and the results of the recovery test are displayed in Table 1. The RSDs of detection were all less than 7.8% and the results showed satisfactory recoveries of 92–109, revealing an acceptable accuracy for detecting in human serum samples. To study the reliability of the proposed immune MEA for human serum samples, the proposed immune MEA was employed to assay five real samples from the Beijing University Cancer Hospital. The enzyme-linked immune sorbent assay (ELISA) was used as a reference method. As shown in the Figure 6, the RSDs of ELISA on the detection of serum samples were 6.2%, 5.6%, 8.2%, 5.3%, and 4.1%, respectively. The RSDs of the results of detecting serum samples by immune MEA ranged from 6.7% to 9.1%. Additionally, the relative deviation of the results detected by the two methods ranged from −7.76% to 5.33% (in Table 2), indicating an acceptable agreement. The test results of these two methods were consistent, indicating that the proposed immune MEA could be potentially applied in detecting the CEA concentration for clinical analysis.

## 3. Materials and Methods

### 3.1. Apparatus and Reagents

The scanning electron microscopy was used to perform the scanning electron microscope (SEM) image of the microelectrode surfaces and energy dispersive X-ray spectroscopy (EDS) for component analysis (S-3500, Hitachi, Tokyo, Japan). The Gamry electrochemical workstation was used to carry out differential pulse voltammetry (DPV) and cyclic voltammetry (CV) (Gamry Reference 600, Gamry Instruments, Warminster, PA, USA). The ELx808 microplate reader was used to read the absorbance. (BioTek Instruments, Inc., headquartered in Winooski, VT, USA).

The (D)-(+)-glucose (≥99.5%) and chitosan (CS, from crab shells, minimum 85% deacetylated) were supplied by Sigma-Aldrich Co., Ltd (St. Louis, MO, USA). Ascorbic acid (AA, 99%) and thionin acetate (THI) were provided by Alfa Aesar. CEA (purified from human fluids) and anti-CEA (CEA monoclonal antibody purified from mouse and the immunogen is human CEA) were supplied by Beijing Key-Bio Biotech Co., Ltd. Uric acid (UA, 99%; Beijing, China) was obtain from Alfa Aesar. Multi-walled carbon nanotubes (purity > 95 wt. %) were supplied by Xianfeng nanomaterials company (Nanjing, China). GA was supplied by Beijing Chemical Reagents Company. Dopamine (DA, ≥99%) was obtained from Acros Organics. Bovine serum albumin (BSA) was provided by LanYi Chemical Reagents Company (Shanghai, China). CEA ELISA kit was provided by Shanghai Linc-Bio Science Co. LTD (Shanghai, China). Human immunoglobulin G (IgG) was provided by Cheng-wen Biological Company (Beijing, China). The phosphate-buffered saline (PBS, 0.1 M Na_2_HPO_4_-NaH_2_PO_4_-KCl, pH 7.4) was provided by a PBS tablet (Sigma, St. Louis, MO, USA). A Michem ultrapure water apparatus was used to purify the water (Michem, Chengdu, China, resistivity 18 MΩ·cm). Clinical serum samples were available from Peking University Cancer Hospital and Institute. All experiments were performed at ambient temperature and all other chemicals were analytical reagent grade without purification.

### 3.2. Fabrication of MEA

**Scheme 2:**
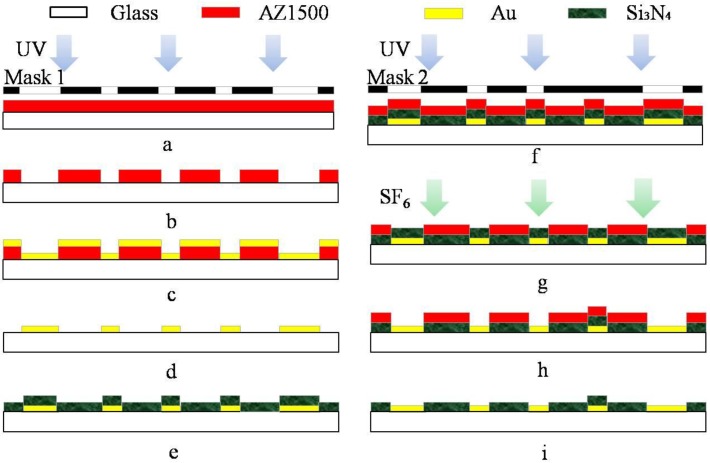
Microelectrode array fabrication process schematic. (**a**) The first photolithography; (**b**) the development of the photoresist; (**c**) magnetron sputtering; (**d**) lift-off process; (**e**) chemical vapor deposition; (**f**) the second photolithography; (**g**) SF_6_ deep reactive ion etcher; (**h**) display the microelectrode sites and pads; (**i**) cleaning step.

As shown in Scheme 2, a standard commercialized glass was firstly washed with acetone, ethanol and deionized (DI) water. The cleaned glass was prepared as the substrate for the fabrication of the MEAs. Before an Au/Cr (200/30 nm) film was grown onto the substrate as the conductive layer by magnetron sputtering (c), the major patterns were displayed by the first photolithographic step using the mask 1 (a,b). The Au/Cr conductive layer was presented following by a lift-off process (d). This conductive layer included electrode lead, pads and 60 microelectrodes. The diameter of circular microelectrode was 20 μm and the space between two microelectrodes was 100 μm. Subsequently, a Si_3_N_4_ (800 nm) was deposited as the insulating layer by plasma chemical vapor deposition (e). Combined with the second photolithographic step using the mask 2 (f), the microelectrode sites and pads were etched for 10 min to display by using an SF_6_ deep reactive ion etcher (SF_6_ DRIE) with a power of 100 W (g,h). Finally, the excess AZ1500 was washed with acetone, ethanol and DI water. The MEAs were manufactured with 60 Au microelectrodes and Si_3_N_4_ insulating layer (i).

### 3.3. Fabrication of CS-MWCNTs-THI Hybrid Film

The redox hybrid film of CS-MWCNTs-THI was firstly deposited to the surface of the bare Au microelectrode by electrochemical reaction. The reaction principles are as follows [29]: (1) chitosan protonated: $\text{CS}\left(\text{Solid}\right){+n\text{H}}^{+}\;\to \;{\left[\text{CS}\right]}^{+}$; (2) chitosan deprotonated: $n{\left[\text{CS}\right]}^{+}{+\;m\text{H}}_{2}O_{2}{\;+\;n\text{e}}^{-}\to \text{CS}↓{+\;m\text{H}}_{2}O$. According to reaction (2), the amino groups of CS became –NH_2_ from –NH_3_^+^ owing to the removal of local H^+^. In addition, the low fluidness of CS resulted in CS forming a stabilizing film to cover the microelectrode. Meanwhile, the catalytic material MWCNTs and electro-active molecular THI were immobilized together to form the hybrid film. The electrochemical deposition was performed in a three-electrode system. The Pt was used as the counter electrode and the reference electrode was provided by Ag|AgCl. The deposition potential was −0.4 V for 300 s. The MEAs were cleaned by oxygen plasma before modification. Before the electrochemical deposition, 300 μL CS (1.0 wt. %) solution containing 2.5 mg MWCNTs, THI (2 mg/mL) and H_2_O_2_ (30 wt. %) were ultrasonicated for 30 min.

### 3.4. Fabrication of the Electrochemical Immune MEAs

After the CS-MWCNTs-THI hybrid film was modified onto the MEAs, the MEAs were immersed into 0.25% GA, which was employed to generate aldehyde group terminals covering the surface of the CS-MWCNTs-THI hybrid film. Then the MEAs were immersed into a 1 μg/mL solution of anti-CEA in PBS (pH 7.4) for 4 h at 37 °C to cross-link antibody and the anti-CEA could be attached by aldehyde group terminals of GA. Finally, the modified immune MEAs were incubated in BSA solution (1.0 wt. %) at 37 °C for about 1 h to cover any possible remaining active sites and avoid the non-specific adsorption.

### 3.5. Electrochemical Measurements

The Gamry workstation was connected with a three-electrode system for the measurement of differential pulse voltammetry (DPV) and cyclic voltammetry (CV). The immune microelectrode was set as the working electrode. Ag|AgCl was used as the reference electrode and the counter electrode was a platinum wire. The immune MEAs were incubated in different CEA concentrations for 30 min at ambient temperature before measurement. The instrumental conditions of DPV experiments were set with a 100 ms pulse time, 25 mV pulse size, 5 mV step size.

### 3.6. The Assay Procedure of ELISA

CEA ELISA kit included 96T and could be used for detection on natural CEA and recombinant CEA. The limit of detection was 0.5 ng/mL and the detection range was from 1 ng/mL to 200 ng/mL. The assay procedure was as follows: (1) set control (zero), standard, test sample wells on the pre-coated plate and recorded their positions respectively. (2) Added 40 μL sample diluent buffer into the control (zero) well and added 40 μL of 0 ng/mL, 4 ng/mL, 20 ng/mL, 100 ng/mL, 200 ng/mL standard solutions into the standard wells. Then added 40 μL sample (serum without dilution) into the sample wells (added the solution into the bottom of each well and avoided bubbles and touching the side wall). (3) Sealed the plate with a cover and incubated 37 °C for 45 min. (4) Removed the cover and discarded the plate content. Clapped the plate on the absorbent filter papers or other absorbent materials and washed plate five times with wash buffer. Added 100 μL HRP-labeled CEA antibody into each well and incubated 37 °C for 45 min. (5) Removed the cover and discarded the plate content. Clapped the plate on the absorbent filter papers or other absorbent materials and washed plate five times with wash buffer. Added 100 μL TMB into each well, covered the plate and incubated at 37 °C in dark for 15 min. (6) Added 50 μL stop solution into all the wells and mixed thoroughly. The color immediately changed from blue into yellow. (7) Read the O.D. absorbance at 450 nm in the microplate reader (ELx808) immediately after adding the stop solution. (8) Repeated the above steps to complete three tests.

For statistical analysis, (the relative absorbance) = (absorbance of each well)-(absorbance of Zero well). The standard curve can consist of the relative absorbance of each standard solution (Y) and the corresponding concentration of the standard solution (X). The CEA concentration of the tested serum samples can be obtained from the standard curve. The microplate reader (ELx808) provided the analyzing software for calculating the CEA concentration of the serum samples.

## 4. Conclusions

A simple label-free electrochemical immune MEA based on CS-MWCNTs-THI hybrid film was developed for detecting CEA. In summary, the highlights of the work are: (1) CS-MWCNTs-THI hybrid film was easily modified onto the MEAs through one-step synthesis by electrochemical deposition. The SEM and EDS results indicated that the good electrochemical property of the immune MEAs was based on the nano-porous structure of the hybrid film and the synergistic effect of MWCNTs and THI. (2) The immune MEA had a wide linear working range for the detection of CEA, from 1 pg/mL to 100 ng/mL with a LOD of 0.5 pg/mL (SNR = 3). (3) The immune MEA had good selectivity, repeatability and good agreement with ELISA for the detection of human serum samples. This novel immune MEA has potential applications for effective early diagnosis, rapid analysis and research on cancer cells and cancer tissue slices.

## Figures and Tables

**Table 1:** Recovery tests of CEA in the human serum samples.

Samples	1	2	3	4	5	6
Initial (ng∙mL^−1^) ^a^	0.39	0.75	0.82	0.45	0.25	0.53
Added (ng∙mL^−1^)	0.50	0.50	1.00	1.00	2.00	2.00
Found (ng∙mL^−1^) ^b^	0.86	1.32	1.81	1.48	2.26	2.51
Relative deviation (%)	7.0	7.8	6.6	7.6	5.1	5.6
Recovery (%)	92.3	109.3	98.7	106.7	104	96.2

^a^ Human serum samples were diluted prior to assay; ^b^ The average value of five successive measurements.

**Table 2:** Assay results of clinical serum samples using the reference and proposed methods.

Serum Samples	1	2	3	4	5
ELISA (g/mL) ^a^	0.78	3.26	1.50	8.17	23.2
Immune MEAs (ng/mL) ^a^	0.81	3.18	1.58	8.43	21.4
Relative deviation (%)	3.85	−2.45	5.33	3.18	−7.76

^a^ The average value of three successive measurements.

## References

[1] Wang R., Chen X., Ma J., Ma Z. (2013). Ultrasensitive detection of carcinoembryonic antigen by a simple label-free immunosensor. Sens. Actuators B.

[2] Liu Z.F., Jin C.J., Yu Z.L., Zhang J., Liu Y., Zhao H.Y., Jia B., Wang F. (2010). Radioimmunotherapy of human colon cancer xenografts with I-131-labeled anti-CEA monoclonal antibody source. Bioconjug. Chem..

[3] Hawkridge A.M., Muddiman D.C. (2009). Mass spectrometry-based biomarker discovery: Toward a global proteome index of individuality. Anal. Chem..

[4] Ferrari M. (2005). Cancer nanotechnology: Opportunities and challenges. Nature Rev. Cancer.

[5] Kong F.Y., Zhu X., Xu M.T., Xu J.J., Chen H.Y. (2011). Gold nanoparticle/DNA/methylene blue nanocomposites for the ultrasensitive electrochemical detection of carcinoembryonic antigen. Anal. Chim. Acta.

[6] Distler M., Pilarsky E., Kersting S., Grützmann R. (2013). Preoperative CEA and CA 19-9 are prognostic markers for survival after curative resection for ductal adenocarcinoma of the pancreas-A retrospective tumor marker prognostic study. Int. J. Surg..

[7] Sun X., Ma Z. (2013). Electrochemical immunosensor based on nanoporpus gold loading thionine for carcinoembryonic antigen. Anal. Chim. Acta.

[8] Tang L.C., Dong C., Ren J. (2010). Highly sensitive homogenous immunoassay of cancer biomarker using silver nanoparticles enhanced fluorescence correlation spectroscopy. Talanta.

[9] Song C.Y., Wang Z.Y., Zhang R.H. (2009). Highly sensitive immunoassay based on Raman reporter-labeled immuno-Au aggregates and SERS-active immune substrate. Biosens. Bioelectron..

[10] Wang Y., Xu H., Luo J., Liu J., Wang L., Fan Y., Cai X. (2016). A Novel Label-Free Microfluidic Paper Immunosensor Based on Graphene Nanocomposites for Highly Sensitive Electrochemical Detection of Carcinoembryonic Antigen. Biosens. Bioelectron..

[11] Shi W.T., Ma Z.F. (2011). A novel label-free amperometric immunosensor for carcinoembryonic antigen based on redox membrane. Biosens. Bioelectron..

[12] Zhang B., Tang D., Liu B., Cui Y., Chen H., Chen G. (2012). Nanogold-functionalized magnetic beads with redox activity for sensitive electrochemical immunoassay of thyroid-stimulating hormone. Anal. Chim. Acta.

[13] Cui Y.L., Chen H.F., Hou L., Zhang B., Liu B.Q., Chen G.N., Tang D.P. (2012). Nanogold–polyaniline–nanogold microspheres-functionalized molecular tags for sensitive electrochemical immunoassay of thyroid-stimulating hormone. Anal. Chim. Acta.

[14] Liu Z., Ma Z. (2013). Fabrication of an ultrasensitive electrochemical immunosensor for CEA based on conducting long-chain polythiols. Biosens. Bioelectron..

[15] Pchelintsev N.A., Vakurov A., Millner P.A. (2009). Simultaneous deposition of Prussian blue and creation of an electrostatic surface for rapid biosensor construction. Sens. Actuators B.

[16] Thipmanee O., Samanman S., Sankoh S., Numnuam A., Limbut W., Kanatharana P., Vilaivan T., Thavarungkul P. (2012). Label-free capacitive DNA sensor using immobilized pyrrolidinyl PNA probe: Effect of the length and terminating head group of the blocking thiols. Biosens. Bioelectron..

[17] Maxwell T., Banu T., Price E., Tharkur J. (2015). Non-cytotoxic quantum dot–chitosan nanogel biosensing probe for potential cancer targeting agent. Nanomaterials.

[18] Zhang L., Liu T., Xiao Y., Yu D., Zhang N. (2015). Hyaluronic acid-chitosan nanoparticles to deliver Gd-DTPA for MR cancer imaging. Nanomaterials.

[19] Pillai C.K.S., Paul W., Sharma C.P. (2009). Chitin and chitosan polymers: Chemistry, solubility and fiber formation. Prog. Polym. Sci..

[20] Wang Z.J., Li M.Y., Zhang Y.J., Yuan J.H., Shen Y.F., Niu L., Ivaska A. (2007). Thionine-interlinked multi-walled carbon nanotube/gold nanoparticle composites. Carbon.

[21] Chen S.H., Yuan R., Chai Y.Q., Xu Y., Min L.G., Li N. (2008). A new antibody immobilization technique based on organic polymers protected Prussian blue nanoparticles and gold colloidal nanoparticles for amperometric immunosensors. Sens. Actuators B.

[22] Jia X., Liu Z., Liu N., Ma Z. (2014). A label-free immunosensor based on graphene nanocomposites for simultaneous multiplexed electrochemical determination of tumor markers. Biosens. Bioelectron..

[23] Han J., Ma J., Ma Z. (2013). One-step synthesis of grapheme oxide–thionine–Au nanocomposites and its application for electrochemical immunosensing. Biosens. Bioelectron..

[24] Hong J., Zhao Y.-X., Xiao B.-L., Moosavi-Movahedi A.A., Ghourchian H., Sheibani N. (2013). Direct electrochemistry of hemoglobin immobilized on a functionalized multi-walled carbon nanotubes and gold nanoparticles nanocomplex-modified glassy carbon electrode. Sensors.

[25] Sun X., Gao Y., Gong Z., Wang X., Zhang Y., Gao J. (2012). An amperometric immunosensor based on multi-walled carbon nanotubes-thionine-chitosan nanocomposite film for chlorpyrifos detection. Sensors.

[26] Jayakumar R., Tokura N., Tamura H. (2007). Sulfated chitin and chitosan as novel biomaterials. Int. J. Biol. Macromol..

[27] Rinaudo M. (2008). Main properties and current applications of some polysaccharides as biomaterials. Polym. Int..

[28] Mourya V.K., Inamdar N.N. (2008). Chitosan-modifications and applications: opportunities galore. React. Funct. Polym..

[29] Wei W., Song Y., Shi W., Lin N., Jiang T., Cai X. (2014). A high sensitivity MEA probe for measuring real time rat brain glucose flux. Biosens. Bioelectron..

[30] Xu H., Wang L., Luo J., Song Y., Liu J., Zhang S., Cai X. (2015). Selective recognition of 5-hydroxytryptamine and dopamine on a multi-walled carbon nanotube-chitosan hybrid film-modified microelectrode array. Sensors.

[31] Rech I., Restelli A., Cova S., Ghioni M., Chiari M., Cretich M. (2004). Microelectronic photosensors for genetic diagnostic Microsystems. Sens. Actuators B.

[32] Bai H., Campo J., Tsai Y. (2013). Sensitive electrochemical thrombin aptasensor based on gold disk microelectrode arrays. Biosens. Bioelectron..

[33] Zhang S., Song Y., Wang M., Zhang Z., Fan X., Song X., Zhuang P., Yue F., Chan P., Cai X. (2016). A silicon based implantable microelectrode array for electrophysiological and dopamine recording from cortex to striatum in the non-human primate brain. Biosens. Bioelectron..

[34] Wang L., Xu H., Song Y., Luo J., Wei W., Xu S., Cai X. (2015). Highly sensitive detection of quantal dopamine secretion from pheochromocytoma cells using neural microelectrode array electrodeposited with polypyrrole graphene. ACS Appl. Mater. Interface.

[35] Wei W., Song Y., Wang L., Zhang S., Luo J., Xu S., Cai X. (2015). An implantable microelectrode array for simultaneous l-glutamate and electrophysiological recordings. Microsyst. Nanoeng..

